# Electroactive Smart Polymers for Biomedical Applications

**DOI:** 10.3390/ma12020277

**Published:** 2019-01-16

**Authors:** Humberto Palza, Paula Andrea Zapata, Carolina Angulo-Pineda

**Affiliations:** 1 Departamento de Ingeniería Química, Biotecnología y Materiales, Facultad de Ciencias Físicas y Matemáticas, Universidad de Chile, 8370456 Santiago, Chile; cangulo@u.uchile.cl; 2 Millenium Nuclei in Soft Smart Mechanical Metamaterials, Universidad de Chile, 8370456 Santiago, Chile; 3 Grupo de Polímeros, Facultad de Química y Biología, Universidad de Santiago de Chile, 8350709 Santiago, Chile; paula.zapata@usach.cl

**Keywords:** Electrically conductive polymers, Electroactive biomaterials, Electrical stimulation, Smart composites, Bioelectric effect, Drug delivery, Artificial muscle

## Abstract

The flexibility in polymer properties has allowed the development of a broad range of materials with electroactivity, such as intrinsically conductive conjugated polymers, percolated conductive composites, and ionic conductive hydrogels. These smart electroactive polymers can be designed to respond rationally under an electric stimulus, triggering outstanding properties suitable for biomedical applications. This review presents a general overview of the potential applications of these electroactive smart polymers in the field of tissue engineering and biomaterials. In particular, details about the ability of these electroactive polymers to: (1) stimulate cells in the context of tissue engineering by providing electrical current; (2) mimic muscles by converting electric energy into mechanical energy through an electromechanical response; (3) deliver drugs by changing their internal configuration under an electrical stimulus; and (4) have antimicrobial behavior due to the conduction of electricity, are discussed.

## 1. Introduction

Polymers have emerged in recent decades as one of the most promising materials in biomedical applications due to their high biocompatibility and degradation/absorption in physiological media [1]. Another key characteristic of polymers is their flexibility in terms of properties and functionalities, allowing their development from bioactive hydrogels to biodegradable thermoplastic polymers [2,3]. The polymer flexibility also includes a broad range of processing techniques, such as: extrusion [4], electro-spinning [5,6], 3D printing [7,8,9], microfluidity [10], and casting [11], among others [5]. Remarkably, by adding/embedding nanoparticles into a polymer matrix, novel nanocomposites can be developed further extending the range of properties and functionalities of polymers. For these reasons, polymers are extensively studied today for tissue engineering [12,13], wound healing [14], artificial muscles [15], and drug delivery [16], among other bio-applications [17].

**Figure 1:**
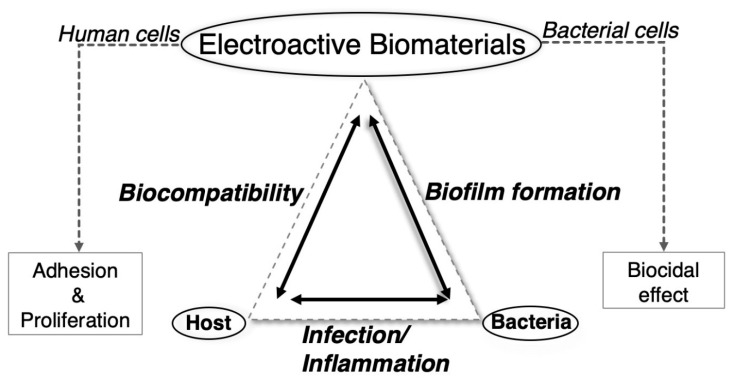
Relationship between electroactive biomaterials and human and bacterial cells in the context of tissue engineering.

Of recent interest in polymer science is the development of smart materials with a rationally designed stimulus/response behavior. In this context, electroactive smart polymer materials are stressed because of their ability to transfer electrons/ions under a specific electric field, having multiple applications in several engineering areas, such as soft robots and sensors [18,19]. The advantages of an electric field as external stimulus, compared to others, is related to the availability of equipment that allows precise control in terms of the current magnitude, the duration of electric pulses, intervals between pulses, etc. However, compared to other functional/smart polymer systems, electroactive smart polymers have been less studied for biomedical applications, despite their multiple applications in tissue engineering [20,21,22]. For instance, these electroactive biomaterials can be applied to obtain adhesion and proliferation of human cells, accelerating the process of regeneration in muscles, organs and bones [23,24,25,26]. They can also be used for smart drug delivery or as artificial muscle systems, both triggered by electric stimuli. Even less studied is the development of biocidal materials based on their electric conductivity despite that today any biomaterial used in tissue engineering must not only be biocompatible in the response of the host (patient) but also active in avoiding the adhesion of bacteria or the formation of biofilms on its surface. Based on the bactericidal effect of electrical stimulation (ES), novel electroactive materials can be produced with the ability to prevent the formation of biofilms and future bacterial infections in the host. Therefore, a polymer able to deliver ES can merge the requirements needed for any biomaterial designed for tissue engineering purposes: to promote cellular adhesion and proliferation while avoiding biofilm formation through a bactericidal effect (see Figure 1). 

**Figure 2:**
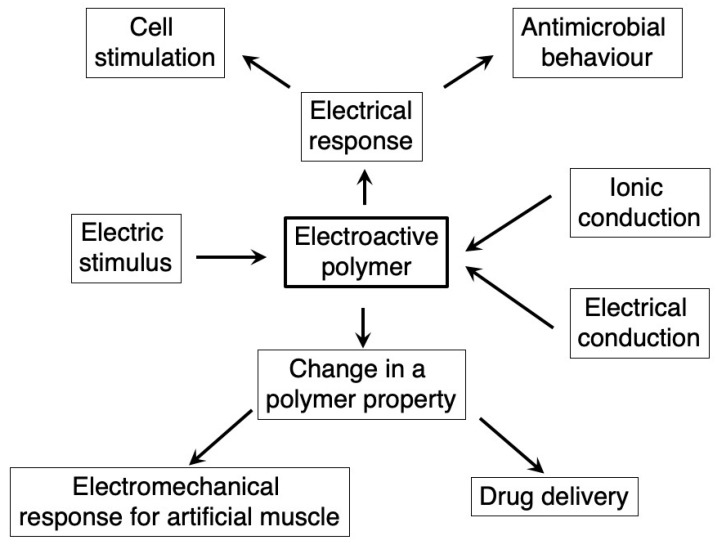
A general overview of electroactive polymers. The mechanism for the specific response to an electric stimulus can be through ionic or electric conduction. These mechanisms can trigger either a direct electric current to the material and the medium producing cell stimulation, or antimicrobial behavior or a change in some polymer properties, producing an electromechanical behavior and specific drug delivery.

In this review, we provide a general overview of the potential of electroactive polymer biomaterials considered as a new generation of smart systems able to respond specifically to an electric field in the context of biomedical applications. These smart systems range from polymers delivering an electric signal to polymers changing some properties under an electric stimulus [27]. The review focuses on the capacity of these electroactive polymers to stimulate: (1) cells in the context of tissue engineering; (2) an electromechanical response for artificial muscles; (3) drug delivery; and (4) antimicrobial mechanisms. From a material point of view, the electroactive polymers include intrinsically conductive polymers, percolated conductive polymer nanocomposites, and ionic conductive hydrogels. A general overview of this review is summarized in Figure 2. For further details about one or more of the above-mentioned electroactive properties or polymers, there are several excellent reviews (for instance, see references [17,21,27,28,29,30,31,32,33]) in which specific information can be obtained for a deeper understanding of an application. 

## 2. Electroactive Conductive Polymers 

**Figure 3:**
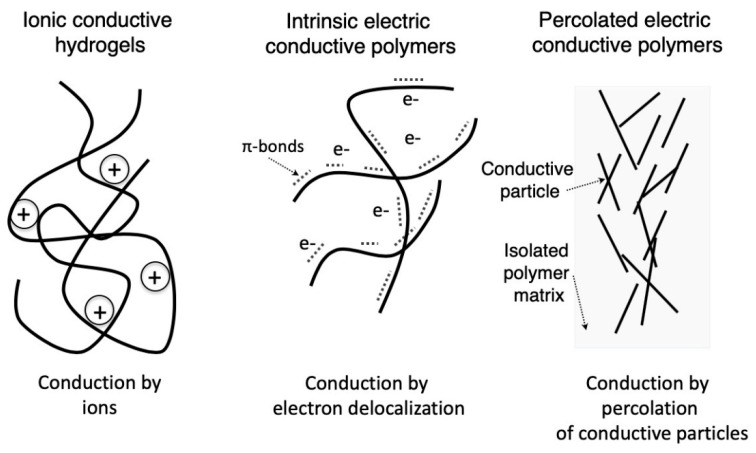
Simplified schematic diagrams showing the different conduction mechanisms of electroactive smart polymers. Ionic polymers present conductivities associated with the presence of polyelectrolytes (left side), while electric conductive polymers can transfer electrons by either an intrinsic mechanism associated with their chemical bonds (middle) or conductive particles percolated into the isolated matrix (right side). See text for details.

**Figure 4:**
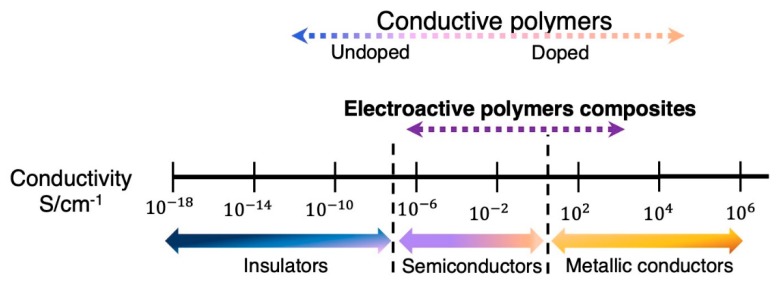
Conductivity range of intrinsically conductive polymers and electroactive conductive composites. Based on reference [29].

Electroactive polymers can be classified according to the mechanism of conduction in ionic conductive polymers and electric conductive polymers. The latter are further classified as intrinsic and extrinsic, based on their mechanism of electron conduction. While ionic conductive polymers present conductivities due to the presence of both ionic groups in their main chain and electrolytes in the medium, electric conductive polymers are conductive due to the high electron mobility arising from either the constitutive bonds between atoms or the presence of conductive particles, as summarized in Figure 3. Regarding electric conductive polymers, different mechanisms of electron conduction produce changes in the achieved conductivity, as summarized in Figure 4.

These conductive materials retain the good properties and flexibility of polymers, so they can be further functionalized for specific applications by optimizing properties such as roughness, porosity, hydrophobicity, conductivity, and degradability [17]. One route for increasing the functionality is to add monomers covalently bonded to functional molecules, although the conductivity is reduced [34]. For biomedical applications, the biocompatibility and biodegradability of electroactive polymers should be further considered. For instance, the application of intrinsically conductive polymers in tissue engineering is limited by the doping concentrations used to obtain electrical conduction, as high concentrations can produce inflammatory responses in tissues [28]. To increase the biocompatibility of conductive polymers, they can be doped with biomolecules or ions, taking advantages of their chemical, electric, and physical structures [17,30]. 

### 2.1. Intrinsically Conductive Polymers

**Figure 5:**
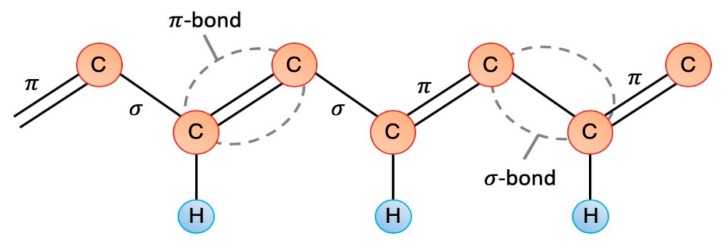
Diagram of the conjugated chain of an intrinsically conductive polymer. Based on reference [35].

Intrinsically conductive polymers present a conductivity mechanism arising from the polymer molecule itself having a conjugated chain that contains localized carbon–carbon single bonds (σ) and less localized carbon–carbon double bonds (π) (see Figure 5). The p-orbitals overlap in the π bonds and give greater electron mobility between atoms, allowing the electrons to move along the polymer chain [27,35]. The conductivity of intrinsic polymers is further based on the incorporation of dopant ions balancing the charge introduced through oxidation (p-doping) or reduction (n-doping) [27]. The dopant introduces a charge carrier by removing/adding electrons from/to the polymer chain and relocalizing them as polarons or bipolarons. The dopants are able to move in or out of the polymer (depending on the polarity) when an electrical potential is applied, disrupting the stable backbone and allowing charge to be passed through the polymer [27]. Intrinsically conductive polymers have attractive properties for use in drug delivery, sensors, electrochemistry, etc. [36,37,38].

Poly[3,4-(ethylenedioxy)thiophene] (PEDOT) [39], polypyrrole (PPy) [40], and polyaniline (PANi) [41] are some of the most widely used intrinsically conductive polymers in tissue engineering scaffolds and biomaterials [17]. However, for biomedical applications, their use is limited mainly because of their poor processability and mechanical properties [36]. The doping of these polymers with long chains can overcome these limitations although it can affect the conductivity of the resulting materials [34,42]. Another solution is to blend the intrinsic conductive polymer with another polymer possessing easier processability in order to obtain a composite with improved mechanical and biocompatibility properties [30]. Such is the case of a 3D coating made of PPy doped with dodecylbenzenesulfonic acid (DBSA) used for electrodes promoting neuronal induction [43]. As discussed above, conductive polymers can be further functionalized with bio-dopants to improve their biocompatibility in medical applications [39]. This method adapts the polymer chains for several applications, improving, for instance, the selectivity/sensitivity of biosensors or the cell-surface interaction [38,40]. Commonly used bio-dopants include glycosaminoglycans such as chondroitin sulfate, hyaluronic acid, and dextran sulfate [30].

### 2.2. Percolated Polymer Composites

By embedding electric conductive particles into a polymer matrix a percolation transition can occur associated with the formation of a continuum network of fillers throughout the polymer. Below the percolation threshold, the conductivity change is negligible, and the conductivity of the composite is equal to that of the polymer. However, the percolation produces a drastic increase of several orders of magnitude in the electric conductivity of the resulting composites. In this case, the polymer matrix is an insulator and the filler is responsible for the electric conduction. In the classical theory of percolation, the conductivity of the composite depends on the filler conductivity, its volume fraction, a critical filler volume fraction at which percolation takes place, and the critical index of conductivity that relates with the dimensionality of the filler [44]. This theory predicts a power-law correlation between these parameters by assuming physical contact between particles. However, electrically conductive polymer composites are more complex systems, as the electric conductivity cannot be fully predicted by this theory [28,45,46]. In polymer composites, the conductive particles are separated by energy barriers (polymer molecules) and the tunnel effect becomes relevant, modifying the percolation model by introducing a tunnel parameter that varies according to the dimensionality of the particle [44]. In this modified percolation model, the composite conductivity depends on the filler conductivity and its volume fraction, but also on the tunnel parameter. Under this model, the effect of the filler on the percolation threshold is rather explained considering the average interparticle distance related to the probability of contact between conductive particles [47], which depends on both the aspect ratio and the particle sizes [48,49]. The introduction of these parameters can explain the different electric behavior found in these polymer composites from a sharp increase in the conductivity reaching a plateau to a broad percolation curve with a growing conductivity [44]. Indeed, although the percolation theory is able to predict some experimental results, currently a different and complementary approach based on the excluded volume theory for percolation transition can explain most of the experimental findings [50]. This theory is based on the evidence that the percolation threshold is not linked to the true volume of the object itself, but rather to its excluded volume [50].

Electrically conductive polymeric composites are currently being developed in order to have light materials that combine the inherent processability of the organic matrix with the electric conductivity of the fillers [50]. Since the first polymer with silver filler was developed for electrically conductive adhesives in 1956, conductive polymer composites have been studied extensively by using gold, palladium, silver, nickel, copper, graphite, and carbon fiber [51]. Among the different fillers, those based on nanoparticles, such as carbon nanotubes (CNT), have emerged as some of the most interesting due to their outstanding properties [52]. In percolated composites, the particle aspect ratio can be considered as one of the most relevant variable, explaining, for instance, that CNT-based composites presented percolation thresholds lower than composites containing metallic particles, carbon black, or carbon fibers, or even some graphite derivatives [53,54]. Actually, the percolation threshold in polymer composites is inversely proportional to their aspect ratio [55]. This is explained by changes in the average inter-particle distance in the composite, with more somewhat spherical structures presenting longer distances. Although nanoparticles render lower percolation transitions than microfillers, their high surface energy produces composites with agglomerated rather than isolated structures, affecting negativelly the electric percolation threshold. It is well known that improving the dispersion state of nanoparticles produces a reduction in the percolation threshold [53,55,56]. For high-aspect-ratio fillers, their alignment is another variable affecting the electric conductivity of polymer/CNT composites [57]. Monte Carlo simulations have confirmed that the conductivity decreases with applied strain, because inter-particle distance increases due to CNT alignment [57]. In general, the percolation transition depends on all these variables in a complex way, and, for instance, an optimal agglomeration and aligment level can be found [57].

The advantage of electric conductive polymer composites is the flexibility of the kind of filler that can be used and other properties emerging from the electric conductivity through the filler. For instance, the current passing through the polymer composite can induce a Joule heating, raising the internal temperature of the polymer composite to above the transition temperature [58,59,60,61].

### 2.3. Conductive Polyelectrolite Hydrogels

Hydrogels are three-dimensional polymeric networks possessing hydrophilic characteristics and high water absorbtion capacities. Due to their high water content, porosity and soft consistency, they can mimmick natural living tissue better than any other class of synthetic biomaterials [62]. Hydrogels can be reversible when the network is formed by molecular entanglements and/or secondary forces such as ionic, H-bonding or hydrophobic forces. If the network is based on covalent bonds joining the macromolecular chains or cross-linking polymers, the hydrogels are permanent. Due to their porous networks and high water content allowing transport of water and small solutes, hydrogels present ionic conductivity, especially in the case of polyelectrolytes, as recently studied by comparing different hydrogels [63]. This ionic conductivity depends on several variables such as polymer polarity, water content, salt/ions, and hydrogel structure. Higher water content increases the ionic conductivity of the hydrogel and leads to a high ion transfer rate [63]. The conductivity of the hydrogel is further controlled by two parameters: the mobility and concentration of ions. In low-concentration electrolyte solutions, the concentration of total ions plays a dominant role in conductivity. In high-salt solutions, the fraction of counterions to the total ions is significantly reduced, so the mobility of the ions becomes the dominant parameter. Ionic hydrogels show higher conductivity than nonionic hydrogels, because cationic and anionic hydrogels have higher concentrations of counterions functioning as charge carriers, leading to high conductivity [63]. Besides the cations and anions of the electrolyte itself, the mobile counterions of the ionic polymers also function as charge carriers, and electrolytes and polymer counterions together contribute to higher ionic conductivity [63].

## 3. Polymers for Tissue Engineering through Electrostimulation of Cells 

### 3.1. Electrostimulation

Living cells use electric fields for several activities associated with: the generation of electromotive force, the control of a specific potential difference, the capacity to control and switch current on and off, and the stored charge. Indeed, an electric voltage exists across the plasma membrane, with the inside of the cell remaining more negative than the outside. Bioelectricity present in the human body plays an integral role in maintaining normal biological functions, such as signaling of the nervous system, muscle contraction and wound healing. During major cellular events like cell division, development, and migration, there is the generation of electric fields [33]. Therefore, a large variety of cell types respond to electrical stimulation, including fibroblasts, osteoblasts, myoblasts, chick embryo dorsal root ganglia, and neural crest cells [33]. 

The inherent bioelectricity present in different cellular events explains the use of electrical stimulation (ES) for tissue repair through either direct current (DC) or alternating current (AC) [22,25]. By applying electric fields, the cell behavior can be modified, including orientation, proliferation, and rate and direction of cell migration, as tested in corneal, epithelial, and vascular cells, among others [17,20,21,31,63,64]. For instance, ES produces electrotaxis or galvanotaxis, the phenomenon by which there is a directional migration of cells in response to the electric field [65,66,67]. There is further evidence showing the great influence of an ES on growth and development of nerve cells, wound healing, and angiogenesis, among other cellular properties, the former being one of the most relevant in this field [68]. In addition, by means of controlled ES, a greater cellular differentiation is achieved; for instance, stem cell differentiation to neurons [33,68,69]. One of the main effect of ES is the opening of ion channels, triggering the production of ions that can be deposited on tissues [70]. This change results in the alteration of ionic fluxes like calcium ions, contributing to cellular locomotion or electrophoretic/electroosmotic effects that cause a redistribution of membrane components [65,67]. The effect of an electrical field is not only valid for cells but also for tissues [71].

### 3.2. Polymers for Electrostimulation of Cells

The construction of scaffolds based on electrically conductive polymers for nerve tissue engineering to enhance the nerve regeneration process have been one of the most studied applications of electroactive polymers [33]. For instance, PC12 cells were seeded on electrochemically synthesized PPy films, producing a ∼91% increase in median neurite length when a positive potential of 100 mV was passed through the PPy for 2 h [72]. Applying electric stimuli to nerve cells through conductive nanofibrous scaffolds of PANi/gelatin enhanced cell proliferation and neurite outgrowth compared with non-stimulated scaffolds can also be achieved [73]. Poly(D,L-lactide-co-ε-caprolactone) membrane coated with PPy and the composite scaffolds increased the proliferation and differentiation of PC12 into neuronal phenotypes as well as sciatic nerve regeneration in rats, showing that they can be used for ES enhancing the neurite outgrowth [74]. These studies demonstrate that cell growth and function can be drastically enhanced at the interface of PPy undergoing ES. 

ES has also been used in fibroblast cells. For instance, conductive biodegradable PPy-Polylactide (PLA) membranes and poly(D,L-lactide)/PPy nanocomposites are able to upregulate the mitochondrial activity of human skin fibroblasts [75]. Under a constant electrical field strength of 100 mV/mm, a greater cell viability was observed than that shown by the non-stimulated cells cultured on the same substrate of identical surface morphology and chemistry. Moreover, electrical field seems to play a more substantial role than does electrical current in modulating the activity of cells cultured on conductive polymeric scaffold. DC applied to nanofibrous scaffolds of PANi and poly(L-lactide- co-ε-caprolactone) enhanced the growth of NIH-3T3 fibroblasts [76]. Electric stimulus in conductive polymers may offer a novel engineering technique to regulate cell adhesion and orientation of bone marrow-derived mesenchymal stem cells (MSCs) and fibroblasts [77].

Regarding the mechanisms behind the effects of an electric potential and/or an electric field on cell activity through an intrinsic conductive polymer, it is speculated that reduction of the polymer (for instance PPy) and electric conduction itself can both affect cells in several ways [34]. For example, the process of neutralization of PPy, under a reducing potential, causes the expulsion of negative ions or the uptake of positive ions from the medium. An uptake of positive ions such as Na^+^ from the medium is speculated to affect several processes, including protein adsorption and the cell cycle. For instance, human Adipose-Derived Mesenchymal Stem Cells (AD-MSCs) attached to PPy/chitosan composite scaffolds and stimulated under DC for 7 days presented a calcium deposition 346% higher than non-stimulated scaffolds [78]. The adsorption of serum proteins, specifically fibronectin, on the electrically conducting polymer can further explain the improved cell behavior under ES as reported in PC12 cells [34,79]. 

Electroactive polymer composites can also be used for tissue regenerating scaffolds, biosensors, and bioapplications, leaving in evidence several potential applications in tissue engineering [29]. For instance, a polymeric composite scaffold of polyacrylonitrile/carbon nanofibers was developed, yielding promising results under ES for applications in nerve tissue regeneration. Electrostimulated cells attached on this conductive scaffold improve neuronal differentiation, and maturation of neural stem cell under 5 V (AC) for 4 h during 7 days [80]. The intracellular and extracellular fluids, which possessed different potentials under ES, produced an extra depolarization, generating these improvements and cell extension [78,79]. Poly(lactic-co-glycolic acid) (PLGA)/CNT electroactive scaffolds were also tested under an electric current (AC) using similar cells with better results than the non-stimulated cell/samples [81]. In particular, an increase in proliferation, differentiation, and growth of long neurites attached to the scaffolds were found under ES in these composite scaffolds [81].

The use of graphene in biomaterials is well known due to its excellent mechanical and electric properties, as well as its biocompatibility with human cells [49,82]. Graphene particles are used as a mechanical support strengthening hydrogels and as electric fillers for percolated conductivity polymers [83]. For instance, electrically conductive graphene hydrogels based on Reduced Graphene Oxide (rGO) and polyacrylamide (PAAm) can be considered as a composite useful for the development of skeletal muscle in soft tissue engineering scaffolds and bioelectrodes. Moreover, ES of myoblasts by the soft electroactive composite can upregulate myogenic gene expressions [83]. Polymer/graphene composite scaffolds can further be designed for cardiac tissue engineering [84]. For instance, Polycaprolactone (PCL) and Graphene composite scaffolds were obtained by an electrospinning technique, producing changes in cardiomyocyte functions and significantly increasing the flux and concentration of Ca^2+^ after ES [84].

Despite the several advantages of electroactive polymers for tissue engineering, some relevant challenges should be addressed in order to continuously improve their behavior in this field. For intrinsic conductive polymers, one the most relevant drawback is the lack of a proper biodegradation among other issues such as poor polymer–cell interactions, the absence of cell interaction sites, hydrophobicity, processability, and mechanical properties [85]. The most common strategy to overcome these issues is to mix the electroactive polymer with another polymer possessing the desired property, such as: PLA, PCL, PLGA, polyurethane (PU), chitosan, gelatin, and collagen, among others, for biodegradation improvements. However, even minimizing the amounts of electroactive polymers in these blends, they are expected to stay in the body. Another route to overcome this limitation is by synthesizing erodible conducting polymers able to have a gradual dissolution [86] or by preparing degradable conductive polymers containing conducting oligomers [85]. For electroactive polymer composites, the potential toxicity of the carbon nanostructures is one the main drawbacks [87]. Moreover, carbon nanomaterials are not biodegradable in general, adding another limitation, although they can be excreted in vivo and cleared from the body once it is no longer needed. The increment in the polymer resistivity after applying an electrical current can further add limitations together with the likely cytotoxicity effect of long-term electrical exposure of cells [27]. 

## 4. Electroactive Polymers for Drug Delivery 

After the discovery more than 50 years ago that hydrophobic and low-molecular-weight drug molecules are able to diffuse through silicone materials at a controlled rate, polymers have been extensively studied for drug delivery systems [88]. The flexibility of polymeric materials can be used to modulate the properties of the materials such as biodegradability and biocompatibility, because of their diversity in chemistry, topology, and dimension. Indeed, polymers show usually improved pharmacokinetics compared to pure small molecule drugs. Polymers are not drugs themselves, and therefore they are designed to provide a passive function as drug carriers, reducing immunogenicity, toxicity, or degradation, while improving circulation time [88]. Relevant in drug delivery is the study of stimuli-responsive polymers mimicking biological systems in the capacity to change under external stimulation [89,90]. These smart polymer biomaterials should present their response within biological conditions. Typical stimuli are temperature [89], pH [91], light [92], electric field [93], and electrolytes [94], among others [95,96,97,98]. The responses triggering the drug release can be: dissolution/precipitation, degradation, change in hydration state, swelling/collapsing, hydrophilic/hydrophobic surface, change in shape, conformational change and micellization. The most important stimuli are pH, temperature, ionic strength, light, and redox potential. However, electric fields can also be a stimuli for drug delivery and today electro-responsive polymers can be considered smart drug carriers [21].

In drug delivery, hydrogels are highlighted because their highly porous structure permits loading of drugs into the gel matrix, subsequently allowing drug release at a rate dependent on the diffusion coefficient of the active molecule through the gel [89,93]. In stimulus/response electroactive hydrogels the final effect of ES on drug release depends to a large extent on how the gel responds to the stimulus, how the drug is released from the gel, and the interactions between the gel network and the drug [99]. The main mechanisms of drug release in these electroactive hydrogels are: (1) forced convection of the drug out of the gel along with syneresed/expelled water due to the electric field [98]; (2) diffusion [100]; (3) electrophoresis of charged drugs [101]; and (4) drug release upon erosion of electro-erodible gels [102]. For charged drugs, the migration of the charged entities towards the electrode bearing an opposite charge should be further considered [103]. The first mechanism is, however, the most important mechanism of drug release in these systems, since under the influence of an electric field, hydrogels generally deswell, causing the movement of solutes out of the gel. In particular, when an electric field is applied, water is syneresed/expelled from the gel, causing the ejection of the drug [98]. When the electric field is removed, the gel absorbs fluid and swells. Thus, upon sequential switching “on” and “off” of the electric field, the gel deswells and swells, following the electric field program [104,105,106]. Three main mechanisms of the electro-induced gel deswelling process exist: (1) the establishment of a stress gradient in the gel; (2) changes in local pH around the electrodes; and (3) electro-osmosis of water coupled with electrophoresis [102]. When diffusion is the major drug release mechanism, electro-induced gel shrinking may inhibit drug release from the gel as the “pores” in the polymer network of the gel become smaller and the pathway for drug movement out of the gel becomes more tortuous. In this case, the application of an electric field stops/reduces drug release from gels. This is especially significant for large drug molecules whose movement out of the gels can be more effectively hindered by a “shrunken” polymeric network [102]. Electro-induced anisotropic gel swelling can also occur when the gel is placed in a fixed position away from the electrodes. In this case, gel expansion occurs when the mobile cations in the aqueous medium migrate towards the cathode, penetrating into the gel network inducing ionization of the carboxyl groups on the gel network that causes the gel on the anode side to swell as the ionized groups become hydrated [107]. These kinds of gels, which swell in response to an electric field (and thus allow drug diffusion out of the gel) may be more appropriate vehicles for electro-controlled release of such large molecules. Finally, pH changes can lead to disruption of the ionic bonds responsible for the gel complex, and for instance the gel surface facing the cathode can dissolve and erode under some conditions. This process triggers drug release.

Intrinsic conductive polymers can also be used for electroactive drug delivery devices, as they can undergo controllable and reversible redox reactions. These reactions alter their redox state, causing simultaneous changes in polymer charge, conductivity, and volume that result in the uptake or expulsion of charged molecules from the bulk of the polymer [107]. By exploiting these changes, the rate of drug release from these conductive polymers can be modified. Anionic drugs can be loaded into the polymers during the oxidative polymerization process or via ion exchange through redox cycling after polymerization. By an electrochemical reduction, anionic molecules can be released [108]. For instance, glutamate anions can be released more than 14 times better from PPy during the application of a reducing voltage, compared to the system without ES. In this case, PPy was prepared with mobile anions that would be released on electric reduction accompanied by polymer contraction (anion-driven actuation), therefore releasing the anionic drug [108]. So the drug release is triggered by reduction and the reincorporation of drug by oxidation. For cationic drug release, when the neutral intrinsic conductive polymer is oxidized, the resulting net positive charge in the polymer repels the drug out of the film. PPy prepared with immobilized anions will incorporate cations on reduction accompanied by swelling (cation driven actuation), and cations can then be released on oxidation. Of interest is mixing intrinsic conductive polymers with hydrogels for the development of electro-conductive hydrogels. In particular, a poly(ethyleneimine) (PEI) and 1-vinylimidazol(VI) polymer blend containing polyacrylic acid (PAA) and poly(vinyl alcohol) (PVA) semi-interpenetrating networks (semi-IPNs) was recently produced for therapeutic electro-responsive drugs [109]. Another electrically active hydrogel was prepared by mixing chitosan-graft-polyaniline copolymer with oxidized dextran (OD) as a cross-linking agent. The copolymer acted as a drug carrier with electrically driven release at a release rate that dramatically increased when an increase in voltage was applied [93]. The electrically driven release of drug molecules from conductive hydrogels has been directly associated with (1) electric field-driven migration of the charged molecules [93] and (2) change in the overall net charge within the polymer upon reduction or oxidation [110].

More complex structures can also be produced using intrinsic conductive polymers for instance those based on nanotubes and microcups. In the former case, biodegradable polymer fibers having the drug were produced by electrospinning, and then the conductive polymer was added on the surface by electrochemical deposition [111]. A local dilation of the tube by the ES promotes mass transport, accounting for the drug release in a desired fashion by ES of the nanotubes. Microcups made of PPy were produced using PLGA polymer as template, with the capacity to control the drug loading/release characteristics [112]. PPy nanoparticles can also be used for drug delivery externally stimulated through a weak and external DC electric field having excellent spatial, temporal, and dosage control [113]. In this case, the conductive polymer was coupled with a temperature-sensitive hydrogel, and the mechanism involved a synergistic process of electrochemical reduction/oxidation and electric-field-driven movement of charged molecules. Recently, electrically responsive micro-reservoirs made of arrays of vertical microtubes were used as support for PPy polymers sealed with PLGA were produced as microcontainers for anti-inflammatory drugs. This system was able to accelerate the cells’ osteogenic differentiation via electrically controlled release of dexamethasone [114].

The electric conductivity of many electroactive polymeric materials used is not high enough to achieve an effective modulation of drug release, leading to the use of more conducting materials (e.g., carbon-based nanomaterials) in polymeric networks as a strategy to enhance the electro-sensitivity of hydrogels. The addition of conductive particles such as CNT can improve the electrically stimulated drug delivery behavior of the intrinsic conductive polymers [115,116]. For instance, a semi-interpenetrating polymer network based on polyethylene oxide and pentaerythritol triacrylate polymers was prepared by electrospinning, and CNT was used to increase the electric sensitivity. The amount of released drug increased under the presence of the conductive particles due to the polymer dissolution under the effects of carbon nanotubes, thereby releasing the drug. A similar tendency was found using an aligned CNT array membrane electrode as a platform for the production of PPy films, showing significant improvement in the controlled release of neurotrophin [117]. Electrospinning was used to prepare poly(vinyl alcohol)/poly(acrylic acid)/multi-walled carbon nanotubes (MWCNTs) nanocomposites where the drug release of nanofibers depended on the electric voltage applied due to the variation of the ionization of functional groups in the polymer matrices [118]. In this context, spherical hybrid hydrogels composed of gelatin with CNT were produced as drug delivery systems for the electro-responsive release of diclofenac sodium salt, where the electrical stimulation increased the drug release associated with a reduction of swelling behavior by built-in osmotic pressure [119]. Electro-responsive hybrid hydrogels can also be produced by this route such as gelatin-coated CNT mixed with acrylamide and polyethylene glycol dimethacrylate as plasticizing and crosslinking monomer, respectively [120]. These composites were highly versatile in modulating the drug delivery of neutral drugs as a function of both nanotube content and voltage magnitude, with drug release being dependent on the balance between electrostatic attractive and repulsive forces and the degree of hydrogel swelling. Another electroresponsive poly(methylacrylic acid)/CNT composite was also reported, presenting controlled drug release upon the On/Off application of an electric field as tested both in vitro and in vivo [121].

The above-mentioned drawbacks of electroactive polymers in tissue engineering are still valid for drug delivery, in particular lack of a proper biodegradation, high hydrophobicity, and poor mechanical behavior [103]. In the particular case of non-biodegradable drug delivery implanted devices, after an initial procedure to administer the device, a second procedure will be required for removal [103]. In addition to these issues, these electroactive polymers will require attachment to an electrode and some electronic circuitry for operating, limiting their use. Another limitation is related to the low levels of drug than can be incorporated and released [103].

## 5. Artificial Muscle Based on Polymer Composites

Artificial muscles can be defined as electromechanical actuators, meaning that they can directly convert electric energy into mechanical energy. They are relevant for a broad range of applications, especially in biomedical engineering, as they can be used in applications such as: microsurgical devices, artificial limbs, or even, in the future, implants like artificial ocular muscles, or hearts [122]. Specific examples are blood vessel (microanastomosis) connectors, tubes that hold open the ear drum (myringotomy tubes), and microvalves for prevention of urinary incontinence [123]. Artificial muscles based on conductive polymer actuators have many advantages for biomedical applications as they (1) can be electrically controlled; (2) have a large strain which is favorable for linear, volumetric, or bending actuators; (3) possess great strength; (4) require low voltage for actuation (1 V or less); (5) can be positioned continuously between minimum and maximum values; (6) work at room/body temperature; (7) can be readily microfabricated and have light weight; and (8) can operate in body fluids [123]. Although different materials are used as artificial muscles, most of them are polymers based on electroactive PPy, ionic metal–polymer composites (IMPCs), hydrogels, or liquid crystal elastomers (LCEs). Today, polymer actuators can even exceed the performance of natural muscle in many respects, making them particularly attractive for use anywhere a muscle-like response is desirable [124]. Each polymer system presents a specific mechanism for the electromechanical actuation and for instance, electronically intrinsic conducting polymers such as PANi and PPy provide one type of high-strain actuator based on dimensional changes produced by electrochemically inserting solvated dopant ions into a conducting-polymer electrode [124]. Dielectric elastomers present actuation through “Maxwell stress” due to the attraction between charges on opposite capacitor electrodes and the repulsion between like charges [125]. The volume change of an electrolyte and electrostatic repulsion can be further used as a mechanism such as in ionic polymer/metal composite actuator. Depending on the conductive mechanisms, these polymers can be divided into two major groups: (1) electroactive polymers (EAPs) such as intrinsic conductive polymers, dielectric elastomer actuators (DEAs), ferroelectric polymers, and liquid crystal elastomers; and (2) ionic EAPs characterized by the presence and movement of ions triggering the actuation [124].

In ionic conducting polymers, an ion is mobile within the matrix and when a positive voltage is applied to a conducting polymer electrode, electrons leave the polymer electrode and anions are attracted to and inserted into the polymer to balance the electric charge, resulting in an expansion. To complete the circuit, a second electrode is used which acts in the opposite direction, expelling ions when it is negatively biased. This inclusion and exclusion of ions can create expansion and contraction on opposite sides of a structure such as a catheter, producing bending [126]. For instance, during oxidation of PPy films, electrons are extracted from the polymer chains, double bonds are rearranged, and positive charges (polarons or bipolarons) are stored along the chains. To maintain the electroneutrality, conformational movements of the chains stimulated by the electrochemical process generate free volume, which is occupied by the counterions and water molecules, producing the film swells. Otherwise, during reduction of the polymer, electrons are injected into the chains and positive charges are compensated. The original structure of the double bonds is restored and counterions and water molecules are expelled towards the solution by the electrochemically stimulated conformational relaxation, promoting a shrinking [127]. Artificial muscles from these conducting polymers are fully reliable Faradaic motors and the movement rate is under linear control of the flowing current and the consumed charge [128]. Design of PPy electroactuators can use a monolithic, bilayered, or trilayered structure, and while monolithic and bilayered implementations are primarily used in applications involving a supporting liquid electrolyte (either aqueous or organic), trilayered ones are employed with an ionic gel electrolyte sandwiched between two PPy films for operation in air. Bending bilayers are one of the most efficient structures transducing reaction that drive from small volume variations in the conductive polymer film to large bending movements [129]. In this case, the second layer is required to translate the volume variation from the polymer film into mechanical stress gradient across the bilayer, producing the macroscopic bending movement. Thus, the second layer is a passive layer that must be bent, although it consumes a fraction of the applied electric energy for bending it. As a result, the muscular energetic efficiency and the angular displacement, for the same consumed charge, decreases [130]. Two layers of the same conducting polymer constituting an asymmetric bilayer muscle can overcome this limitation as one PPy is expected to swell during oxidation by entrance of anions pushing the bending movement while the second layer must shrink during oxidation (simultaneously) by expulsion of cations pulling the bending movement. The improvement arising from the asymmetric bilayer can be seven and four times that of the two layer artificial muscles [128]. A cooperative electro-chemo-mechanical actuation of each of the individual layers occurs in each asymmetric bilayer. 

A different kind of material broadly used for actuators in artificial muscles is the family of ion-exchange polymer–metal composites (IPMCs) showing large deformation in the presence of a low applied voltage. IPMCs consist of a solvent swollen ion-exchange polymer membrane laminated between two thin flexible metal (typically percolated Pt nanoparticles or Au) or carbon-based electrodes [131]. The mechanism in IPMCs is based on the characteristic of polyelectrolytes to possess ionizable groups on their molecular backbone that can be dissociated to obtain a net charge in a variety of solvent media. Therefore, the capacity of these polymers to interact with externally applied fields as well as their own internal field triggers the electromechanical deformation of such polyelectrolyte. For instance, polyelectrolytes filled with liquid containing ions can also deform under an external electric field due to the electrophoretic migration of such ions inside the structure [132]. An IPMC bends toward the anode if it is cationic under the influence of an imposed electric potential, and can oscillate in response to an alternating input voltage. Furthermore, the appearance of water at the surface of the expansion side and the disappearance of water on the contraction side occur near the electrodes, meaning that charged particles drag water molecules parasitically with them when they are electrophoretically transported within the IPMC. Therefore, the imposition of an electric field produces an electrophoretic dynamic migration of the mobile cations that are conjugated with the polymeric anions that can result in a local deformation of the material [132]. These composites are produced in two steps: (1) a compositing process to metallize the inner surface of the polymer by a chemical-reduction where the metallic particles are concentrated predominantly near the interface boundaries; and (2) a surface electroding process in which multiple reducing agents are introduced, and where the original roughened surface disappears [133]. The particles improve the conductivity between the polymer and the electrodes. 

Another approach for artificial muscle is based on dielectric elastomer actuators that are essentially compliant variable capacitors consisting of a thin elastomeric film coated on both sides with compliant electrodes [131]. When an electric field is applied across the electrodes, the electrostatic attraction between the opposite charges on opposing electrode and the repulsion of the like charges on each electrode generate stress on the film causing it to contract in thickness and expand in area. This concept extends toward the construction of flexible dielectric elastomers by the production of a soft dielectric sandwiched between two soft conductors that are subject to a voltage producing electric charges of the opposite polarities accumulate on the faces of the dielectric, causing the dielectric to reduce thickness and expand area [134]. This approach can be further extended to soft robots, where an encapsulated hydrogel serves as an ionically conductive electrode and surrounding tap water can be used as the other electrode [135]. When a voltage is applied the positive and negative charges accumulate on both sides of the dielectric elastomer, inducing Maxwell stress that deforms the membranes. The net effect is a reduction of the body’s curvature, corresponding to the actuated state. The resulting strain in these systems is proportional to the quadratic of the applied voltage and the material electrical strength [136]. Indeed, by increasing the electrical breakdown strength, lager range of input operating voltages and reduced probability of material degradation can be obtained. Notably, the electrical breakdown and the dielectric losses can be changed by controlling processing parameters of the polymer synthesis and fabrication procedure as recently shown for Poly(vinylidenefluoride–trifluoroethylene–chlorotrifluoroethylene) terpolymer [136]. 

A much less studied material for electro-actuators are percolated electric conductive polymer composites, where the mechanism is based on heating the polymer by the joule effect due to the current passing through the conductive paths. This heating produces an observable expansion of the composites and the buckling of the device when the boundaries are restricted [137]. Although these composites can present low volume changes at high voltages, it depends on the materials used and a chitosan/CNT composite can present larger electromechanical actuations [138]. Recently, environmentally friendly electrothermal bimetallic actuators based on waterborne polyurethane and a silicone rubber matrix filler with CNT presented an improved behavior. Under 7 V AC, the actuator achieved a bending displacement up to 28 mm, which is greater than most of other electrothermal actuators reported [137]. 

Despite the potentiality of artificial muscles based on electroactive polymers and hydrogels, they present a major drawback related with the small electrochemical stability window of aqueous electrolytes (≈1.23 V) [139]. Beyond this window, electrolysis of water can lead to catastrophe due to hydrogen and oxygen evolution reactions at the electrodes. Indeed, although these systems can be stable in air, they exhibit slow response time. Moreover, it can be a drift in the bending amplitude which may require correction by a feedback-loop control system. In hydrogels, the main drawback relates with the relatively slow response time as well as chemical stability and performance degradation over time [139]. 

## 6. Antimicrobial and Antifouling Polymers Based on Electrical Stimulation

### 6.1. Microbial Infections and Biofouling

**Figure 6:**
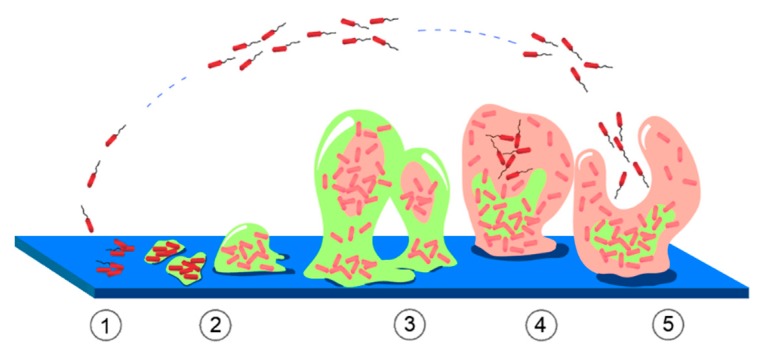
States of biofilm (or biofouling) formation on a material surface (based on reference [140]) State 1: reversible anchoring of bacterial colonies; State 2: bacterial colonies are irreversibly anchored to the surface; State 3: maturation; State 4: the maturation phase is completed and State 5: the bacterial colonies begin to move again, dispersing in microcolonies.

Microorganisms are present at all time in different environments, so it is necessary for the design of any kind of biomaterials to consider their antimicrobial properties [140]. Biofouling is the formation of a microbial consortium which contributes to the development of biofilms capable of adhering to the surface of materials, facilitating the adhesion of other microorganisms on wet surfaces. The development of biofilms on different surfaces is a problem that affects several materials in applications such as food, drinking water quality, and medicines, among others [141]. The bacterial colonies on the surface of a biomaterial, which are highly resistant to antibiotic treatments, are difficult to be eliminated by conventional methods [142], leading to a chronic inflammatory response. Once formed, biofilms cause serious and even fatal clinical complications. In biomedical applications, bacterial infections can cause tissue destruction, premature device failure, and the spread of infection to other areas [143,144]. For instance, bone implants are always associated with risks of bacterial infection that leads to implant failure or, in critical cases, amputation or death of the patient [144,145]. In contact with the eye lens this process causes serious eye infections [146]. Other examples of fouling formation are in catheter-associated urinary tract infections [147,148], and dental implants cause periodontal diseases and gingivitis [144]. Therefore, it is of great importance to eradicate biofilm formation avoiding the reversible anchoring of bacterial colonies [149]. The formation of bacterial films on a surface can be classified as follows: State 1—reversible anchoring of bacterial colonies; State 2—bacterial colonies irreversibly anchored to the surface, losing the flagella that give spatial mobility; State 3—beginning of the first maturation stage; State 4—completion of the maturation phase; and State 5—movement of bacterial colonies and dispersal into microcolonies [140]. These states are summarized in Figure 6. The different strategies for the control of biofilms are still under discussion, although some of them are: inhibit microbial adhesion on the surface, interfere with the surface by molecules that modulate the development of the biofilm, and the dissociation of the biofilm matrix [140].

### 6.2. Electrical Stimulation as an Antimicrobial Method 

ES has been applied to promote the inactivation of different biofilms and bacterial strains, such as S. aureus, Pseudomonas, and E. coli on different metals and amorphous carbon substrates, among other types of electroactive materials [150,151]. Because all naturally occurring surfaces, including those of bacterial cells, are generally negatively charged, the electrostatic force between bacteria and a biomaterial surface is repulsive. These repulsive forces can be enhanced by application of an electric current, thereby increasing the negative charge and consequently the repulsive force [152]. Therefore, this electrostatic repulsion between the resulting electrically charged material surface and biofoulants such as soluble microbial product molecules and extracellular polymeric substances which are negatively charged, and microbial cells can facilitate their removal [153]. An electric current can further enhance the activities of antimicrobial agents such as aminoglycosides, quinolones, and oxytetracycline against Pseudomonas aeruginosa, Klebsiella pneumoniae, Staphylococcus epidermidis, Escherichia coli, and Streptococcus gordonii biofilms, a phenomenon referred to as the bioelectric effect [154]. This effect can be related to pH modification, the production and transportation of antimicrobial agents into the biofilm by an electrophoretic process, the genesis of additional biocidal ions, or hyperoxygenation [155]. The bioelectric effect has been studied mainly in infections associated with metal prostheses, although studies have also been conducted to the treatment of infections in the auditory canals, through non-invasive transcutaneous or minimally invasive applications such as subcutaneous [154].

In addition to the mechanism based on electrostatic repulsion, there are others not yet fully understood [148,149]. For instance, the electric field causes an increase in the permeability of the cell membrane, causing electropermeabilization or irreversible electroporation and the production of reactive oxygen species (ROS) [156,157,158,159,160,161,162,163,164]. This electrolytic damage of the internal cell membranes generates an irreversible loss of the semipermeable barrier function, the release of intracellular content, a loss of motility, and synthesis of some enzymes such as lactate dehydrogenase and trypsin [160]. The effects of electroporation produced by low electric fields (1.5–20 V/cm) promotes biocidal action in the different existing biofilms [160,161,162]. Free radicals and ROS are generated as hydrogen peroxide and reactive nitrogen species (RNS) at low electric field and low current [155,164]. Moreover, electric current, even at a low intensity, can cause an increase of hydrogen ion concentration inside the cytoplasm and disorganization of membrane functionality, causing the alteration of cells. It has been demonstrated that the use of AC causes the inhibition of yeast cell metabolism because it induces the migration of electrons from the cell to the graphite electrode and the accumulation of H^+^ ions in the cell, thereby modifying the membrane potential [160].

### 6.3. Electroactive Polymers as Antimicrobial and Antifouling Materials

Despite the relevance of electric field to avoid biofouling, its use in electroactive antimicrobial polymers for biomedical applications has been barely reported. For instance, modified PPy membranes coated with graphene derivatives were produced to enhance their electric conductivity and improve biofouling suppression because of higher electrostatic repulsions [153]. In general, most of the research has focused on antifouling membranes for bioreactors. The mechanisms of fouling prevention and cleaning with conductive membranes are also mainly based on electrostatic interactions or electrochemical redox reactions on the membrane surface [165]. For instance, during filtration of charged macromolecules and particles, the charged conducting membrane pushes back the foulants due to the electrostatic effect, and this reduces membrane fouling. In electrochemical fouling, the membrane acts either as the electrode where direct or indirect oxidation of foulants takes place on the membrane surface or at the electrode, where foulants are removed via bubble generation on the surface [165]. In this context, intrinsic conductive polymers are able to show antimicrobial behavior without any external electric stimulus due to the oxidative stress that these polymers can generate on the bacterial cells, suppressing the formation of the bacterial cell wall [165]. Nanocomposites of PANi with zinc oxide (ZnO) nanorods, and epoxy resins with PANi, showed excellent antifouling properties [165,166] By developing an electrically conductive membranes through a graphene (Gr) and PANi coating doped with phytic acid (PA) on polyester filter cloth, a membrane with good conductivity was obtained, presenting excellent antifouling properties. The membrane with a higher conductivity had better antifouling property [166]. 

One of the first reports about polymer composites for electric antimicrobial effect in biomedical applications used carbon particles where two modified catheters were placed vertically in a nutrient agar plate and connected to an electric device with one catheter acting as a cathode and the other as an anode [167]. The bactericidal activity possessed by negatively charged electroconducting polymers was explained by the establishment of electrostatic repulsions between the negatively charged bacterial cell wall and the polymer [168]. Recently, Arriagada et al., 2018 [169] achieved 100% antimicrobial activity by applying 9V by means of an electroactive composite based on Poly(lactic acid) (PLA) with Thermally Reduced Graphene Oxide particles (TrGO). The results are attributed to the electrostatic effect and the transfer of electrons in conductive materials under an electric current, which causes the death bacteria attached to the electroactive materials [169]. Future research should focus on polymeric compounds capable of eradicating in the early states of microorganisms attaching to surfaces through new smart electroactive biomaterials [170]. In Zhang et al., 2014 [170] Polypyrrol (PPy)/chitosan films with a synergic effect of DC current and gentamycin treatment against biofilm bacterial were fabricated, and they were able to produce biofilm disruption by compromising the integrity of the cell wall by an autolysis-induced cell disruption, i.e., through the action of enzymes produced under the applied DC [170].

In hydrogels the effect of an electric current on the bacterial growth has also barely been reported. For instance, a DC electric field was used as a practical nonthermal procedure to reduce or modify the microbial distribution in alginate and agarose gel beads. The viability of bacteria entrapped in the beads decreases as the field intensity and duration of electric field increase [171].

## 7. Conclusions

The flexibility of polymers makes possible the development not only of highly compatible and degradable biomaterials, but also a broad set of conductive materials such as: intrinsically electric conductive polymers, percolated electric conductive composites, and ionic conductive hydrogels. This unique flexibility of polymers can be used for the design of electroactive materials for specific biomedical applications such as ES of cells; drug delivery; artificial muscles; and antimicrobial materials. While the use of ES in conductive polymers has been well documented for drug delivery and artificial muscles, more research should take place regarding the potential use of these smart polymeric materials for cell proliferation and antimicrobial scaffolds. For instance, additive manufacturing can extend the range of possibilities for designing electroactive scaffolds that would certainly impact the applications of electroactive polymers.
